# Analogous comparison of registered brand name drugs of tablets and capsules commercially available in Thailand: A retrospective study

**DOI:** 10.1371/journal.pone.0276321

**Published:** 2022-10-19

**Authors:** Jintana Napaporn, Pitchaporn Buakaew, Patarawat Suksakornthanawat, Saksit Sripa, Peerawat Jinatongthai, Teeraporn Supapaan

**Affiliations:** 1 Faculty of Pharmaceutical Sciences, Ubon Ratchathani University, Ubon Ratchathani, Thailand; 2 Sisaket Hospital, Mueng district, Srisaket, Thailand; 3 Namphong Hospital, Namphong district, Khonkaen, Thailand; University of Porto, PORTUGAL

## Abstract

Drug name confusion or similar product packaging and labeling, also known as “look-alike, sound-alike” (LASA) medication error, is one of the most problematic causes of prescribing and dispensing errors. Therefore, this study aimed to compare the similarity of registered brand name drugs of tablets and capsules that are commercially available in Thailand to estimate the magnitude of LASA medication errors. Analogous comparisons of brand names using similarity in orthography (written forms with identical letters) were analyzed retrospectively. Tablets and capsules commercially available in Thailand and registered with the Bureau of Drug Administration, Food and Drug Administration (FDA) in 2012 as “dangerous drugs” and “specially controlled drugs” for humans and animals were included in this study. Descriptive statistics, including frequencies and percentages, were used in this study. The analogous comparison of brand name orthography was scrutinized, and the results revealed 1,668 brand names, which were categorized into three genres as follows:

1) Single brand names from a single manufacturer having the same active pharmaceutical ingredient (API) with numerous registration numbers (1,049 names, 62.89% of the total similarity results)

2) Single brand names from different manufacturers having the same API and possessing several registration numbers (615 names, 36.87% of the total similarity results)

3) Single brand names from different manufacturers with diverse APIs (four brand names, 0.24% of the total similarity results).

Analogous results revealed that numerous identical brand names could be derived from the same manufacturers, APIs, dosage strengths, or otherwise. The results of this study recommend improvement on product registration to better ensure patient safety in the future.

## Introduction

Patient safety, defined as the liberation of patients from unnecessary or potential harm associated with healthcare services [1], is a major concern of the World Health Organization (WHO). Evidence suggests that adverse drug events occur worldwide, some of which can be prevented. Thai FDA criteria for spontaneous adverse event report composes of 5 parts; patient information e.g., patient ID, patient initials, age, weight, underlying disease / other relevant conditions, patient type, gender, race, and history of allergies; health product information e.g., type of health product, product name, dose and administration, starting date, discontinuing date, disease/reason for use, and source of product; adverse event information e.g., characteristics of adverse events, date of onset, laboratory findings and physical evidence, seriousness and outcome of adverse event; source of event and reporter information; cause of event. Importantly, health product information must be specified generic name, trade name and dosage form. However most of ADR reports appear to be incomplete due to missing trade name to identify specific products. Moreover, the lack of information might come from hospitals and private clinics providing some medication as pre-packs products, dispensed with inadequate medication label (no specific brand name, only generic name).

Therefore, the WHO introduced a regional strategy for patient safety in the Southeast Asia Region (2016–2025) to ensure the quality and safety of the healthcare system. Medication safety is a strategic objective to promote global patient safety campaigns and strengthen patient safety in all healthcare systems [1]. Medication safety strategies focus on minimizing medication errors, especially on “look-alike, sound-alike” (LASA) drugs, which cause unintentional mistakes in drug prescribing, dispensing, and administration [2–6].

LASA drug names have been comprehensively reported by the Joint Commission, United States Pharmacopoeia MED-MARX drug error-reporting system [7,8]. LASA data include not only identical brand names and international nonproprietary names (INN), but also identical packaging and labeling [9]. In Thailand, commercially available drug products can cause confusion and medication errors due to similar packaging, poor design, or unsuitable labeling, especially when generic drugs are produced by different manufacturers. An inadequate regulatory system may also be partially responsible for the failure to prevent drug product confusions or LASA errors.

LASA errors have been increasingly recognized as the leading cause of medication errors in Thailand hospitals [10,11]. These errors can be derived from labels and packaging of medications from the same pharmaceutical company and similar brand names [10]. In 2017, the Ministry of Public Health of Thailand initiated the National Patient and Personnel (2P) Safety Policy, striving for “Safety to all, Happy for all’ goals [12]. LASA drugs are regarded as a critical concern in Thailand’s national patient safety policy; hence, strategies to prevent LASA drug name errors have been implemented.

LASA drug name errors have been attributed to brand name confusion [11,13,14]. As of October 2012, 15,005 brand names of tablets and capsules were registered with the Bureau of Drug Administration, the Thailand Food and Drug Administration (FDA) as “dangerous drugs” and “specially controlled drugs” for humans and animals. LASA drugs involve medications that are visually similar in physical appearance or packaging and names with spelling similarities and/or similar phonetics. Similar phonetics could be a comparison of generic names with generic names, generic names with brand names, or brand names with brand names. However, no studies or reports have focused on the similarity of registered brand names of tablets and capsules. Therefore, this study aimed to compare the similarity of registered brand names of tablets and capsules commercially available in Thailand, to estimate the magnitude of LASA medication. To the best of our knowledge, this study is the first to analyze the similarity of registered brand names of tablets and capsules commercially available in Thailand.

## Materials and methods

### Outline of the study

This retrospective comparison study was conducted by researchers from the Faculty of Pharmaceutical Sciences at Ubon Ratchathani University, Thailand. This research was a descriptive study based upon analyzing the legitimate data of registered brand names of “dangerous drugs” and “specially controlled drugs” for humans and animals, obtained from the Bureau of Drug Administration, the Thailand FDA. This study had two main objectives: 1) to evaluate the analogy of brand names of tablets and capsules commercially available in Thailand that are registered with Thailand FDA in 2012 as “dangerous drugs” and “specially controlled drugs” for humans and animals; and 2) to state the immensity of LASA name errors to provide a recommendation for regulatory authorities accountable for drug registration to ensure National 2P Safety Policy goals.

### Study protocol

Secondary data of registered brand names of “dangerous drugs” and “specially controlled drugs” for humans and animals in 2012, utilized in this study, were obtained from the Bureau of Drug Administration, the Thailand FDA. Permission to use the secondary data was granted. Therefore, the ethics committee waived consent. The Ubon Ratchathani University Ethics Committee for Human Research approved this study (UBU-REC-23/2561).

### Procedure

As of October 2012, there were 15,005 tablets and capsules commercially available in Thailand and registered as “dangerous drugs” and “specially controlled drugs” for humans and animals with the Bureau of Drug Administration, the Thai FDA. The minimal underlying data set of this study is published and can be searched at https://drugiden.ubu.ac.th/. Considering tablets and capsules have been two most registered dosage forms (≥ 60% of drugs available in the market), analogous comparisons were queried exclusively for tablets and capsules. Registered brand names of tablets and capsules were then orthographically compared with all cases. In this study, orthographic similarity was defined as the similarity of registered brand names (letter by letter). Registered brand names were sorted in alphabetical order and their similarities or differences were marked by content analysis using Microsoft Excel. Similarity results were validated by an individual researcher, and a triangulation technique for validation by peer reviews was used to generate an arbitrary analogous comparison [15,16].

### Data analysis

Data were analyzed using the content analysis method. Data analysis have been conducted rigorously using a triangulation technique for validation by peer reviews. The registered brand names of tablets and capsules were counted and compared for alphabetical identification [17]. The researchers classified the acquired identical brand names on account of registration number, manufacturer detail, APIs, dosage forms, and strength. Descriptive statistics, including frequencies and percentages, were used in this study.

## Results

**Fig 1:**
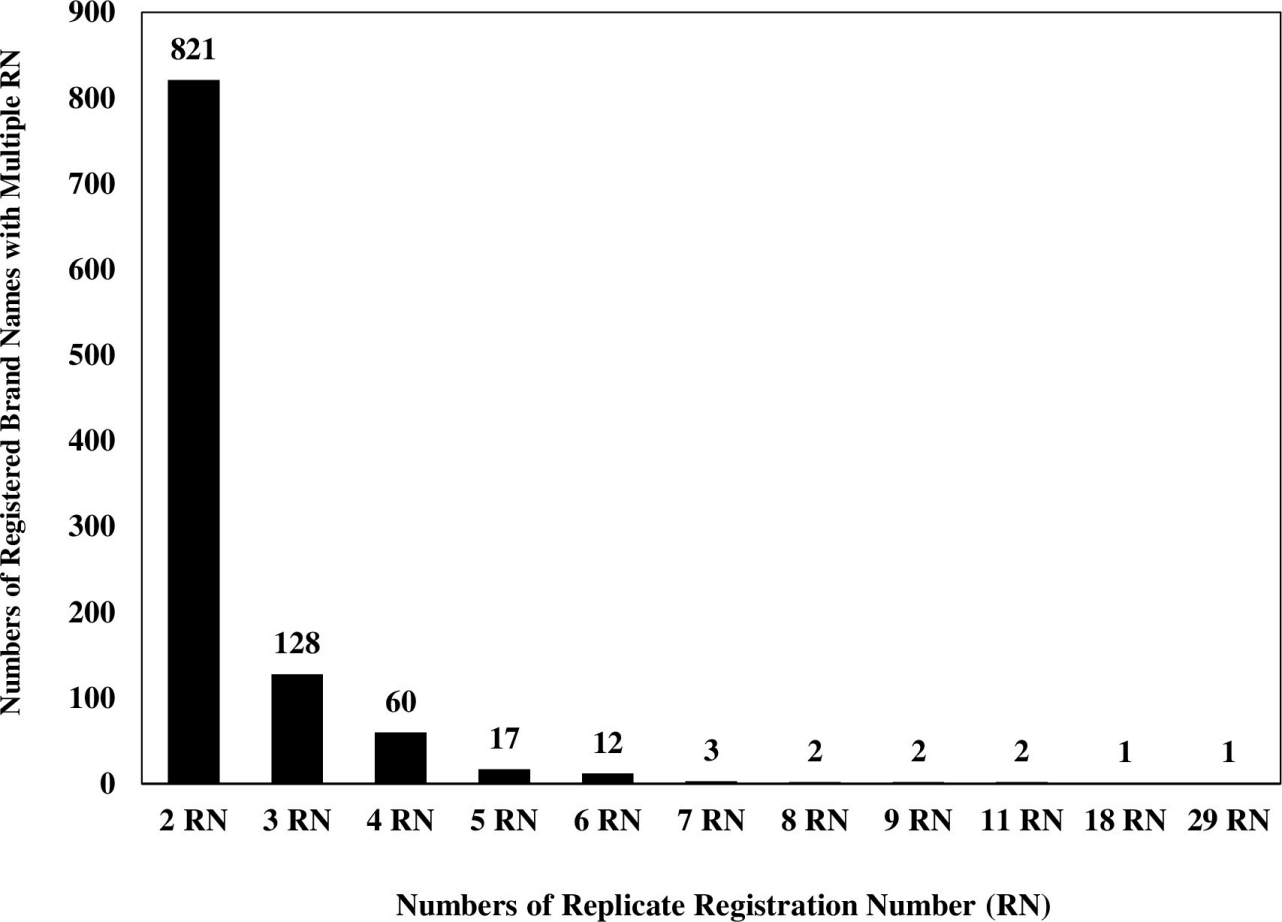
Analogy results of single brand names from single manufacturer having the same active pharmaceutical ingredients (APIs) with numerous registration numbers.

**Fig 2:**
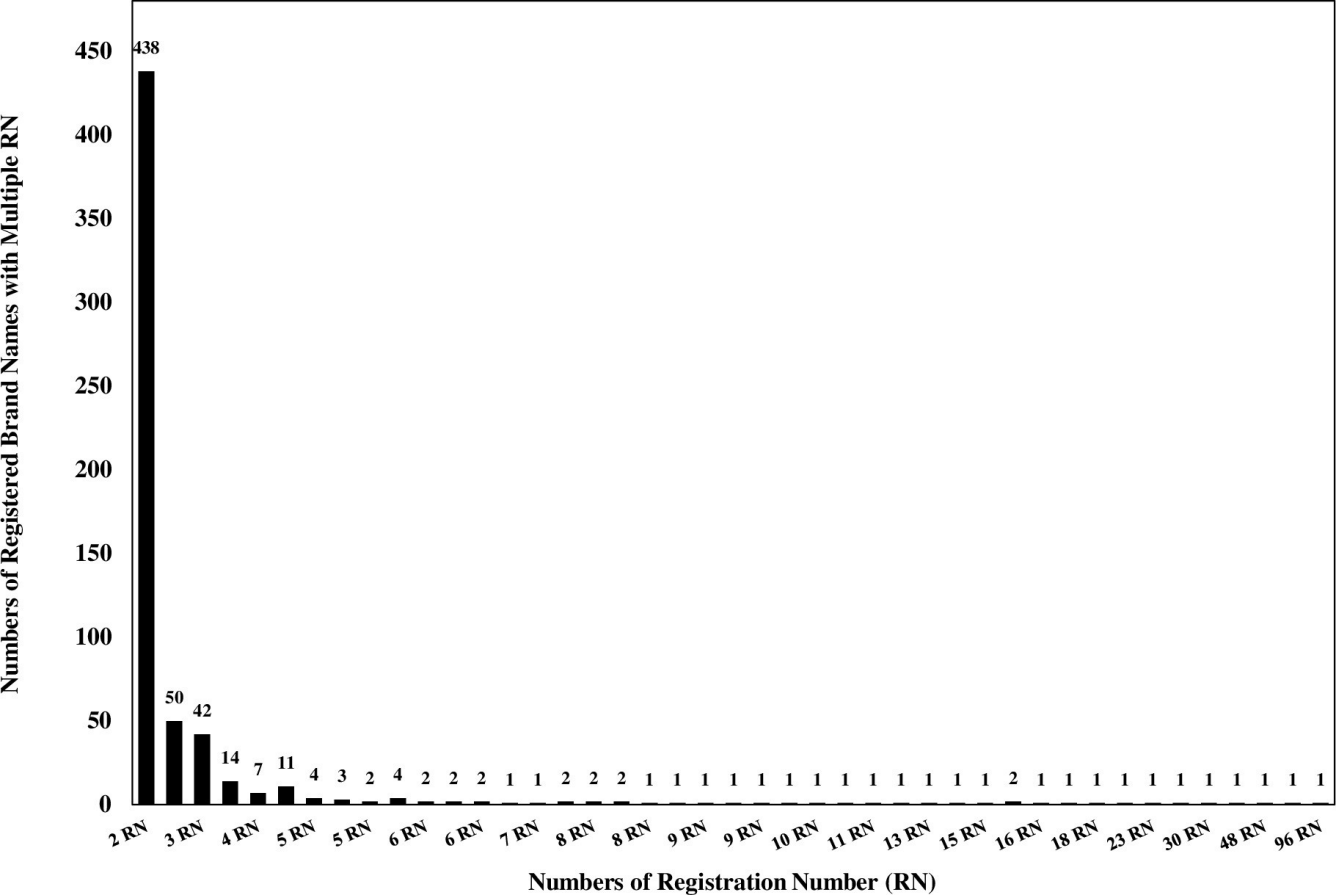
Analogy results of single brand names from different manufacturers having the same APIs and possessed several registration numbers.

In 2012, there were 15,005 registered “dangerous drug” and “specially controlled drug” tablets and capsules for humans and animals in Thailand. The pair-wise similarity of the registered tablet and capsule brand names was investigated (letter by letter) by an individual researcher, and an arbitrary analogous comparison was generated by data validation via peer reviews. Analogous results demonstrate that 1,668 registered brand names possess more than one registration number. The classification of analogous results based on registration number, manufacturer detail, APIs, dosage forms, and strength has been categorized into three genres as follows:

1. Single brand names from a single manufacturer having the same API with numerous registration numbers (1,049 registered names; 62.89% of the total similarity results). Such registered brand names could depict a range from 2 to 29 registration numbers (Fig 1). Registered brand names with two registration numbers were recognized to have the highest frequency, with 821 pairs. Cemol^®^ (paracetamol 500 mg) had the most registration numbers (i.e., 29) from one manufacturer. Among various APIs, paracetamol, prednisolone, amoxicillin, chlorpheniramine maleate, and diclofenac were the five most frequently reported APIs with multiple registration numbers.
2. The second genre of analogy was single brand names from different manufacturers having the same APIs and possessing several registration numbers. The results illustrated that 615 brand names (36.87% of the total similarity results) had fallen into this genre (Fig 2). A total of 438 registered brand names appeared to have the same API, while each brand name had two different registration numbers and was manufactured by different pharmaceutical companies. Paracetamol tablet^®^ (500 mg) was the foremost brand name of APIs manufactured by 33 pharmaceutical companies, with 96 registration numbers. Dexamethasone tablet^®^(0.5 mg) manufactured by 22 pharmaceutical companies with 57 registration numbers was the second most frequently replicated brand name. Furthermore, Prednisolone tablet^®^ (5 mg) was ranked the third most frequently replicated brand name with 48 registration numbers (from 23 manufacturers).
3. The last genre was a single brand name from different manufacturers with diverse APIs. This similarity was detected seldomly (0.24% of the total similarity results). There were four brand names (Myogesic^®^, Nizol^®^, Gemzil^®^, and Ferromin^®^) that represented totally different APIs, manufacturers, and registration numbers (Table 1).

**Table 1:** Lists of registered brand names in single brand names from different manufacturers with diverse APIs genre.

Registered Brand Name	APIs	
	Registration number #1	Registration number #2
Myogesic^®^	Paracetamol 450 mg and orphenadrine citrate 35 mg	Tolperisone HCl 50 mg
Nizol^®^	Ketoconazole 200 mg	Clotrimazole 100 mg
Gemzil^®^	Gemfibrozil 300 mg	Captopril 25 mg
Ferromin^®^	Ferrous fumarate 200 mg	Ferrous gluconate 600 mg

## Discussion

To the best of our knowledge, this retrospective study is the first to analyze the similarity of brand names of tablets and capsules registered as “dangerous drugs” and “specially controlled drugs” (2012) in Thailand. Thailand FDA had defined “dangerous drugs” as medicines that can be purchased without a prescription at a community pharmacy, such as paracetamol, chlorpheniramine, antibiotics, oral contraceptives, and antihypertensive medications; and “specially controlled drugs” as medicines that Registered brand names were inspected for identical orthography. The analogy results had illustrated 1,668 identical brand names, and each brand name occupied more than one registration number. Those identical brand names could derive from the same manufacturers, same APIs, same dosage strengths, or otherwise. Therefore, 3 genres were designated based on the manufacturer, dosage strength, and APIs.

The first genre, single brand names from single manufacturer having the same active pharmaceutical ingredients (APIs) with numerous registration numbers, contained 1,049 registered names. The identical names with 2 registration number were found 821 pairs (total of 1,642 brand names, 10.96% of registered tablets and capsules available in the market at that time). This number indicated that revision of drug registration should be reconsidered for better efficiently monitor and discontinue the obsolete formula from the market. If one registration number was outdated, it should be terminated and replaced with a revised version. The registration process should be designed to encourage both pharmaceutical manufacturers and regulatory authorities to determine whether the similarity in brand names are acceptable on the market. However, if pharmaceutical manufacturers decide to pursue more than one registration number for one formulation, brand name and physical character distinction of the product should be apparent [4,5,8,18]. Theoretically, if one API (single strength) from one manufacturer has one registration number, redundancy in registration numbers can be reduced to 1,498. This may facilitate the Thai FDA’s anticipation of the actual number of registered drugs available on the market and their usage.

Prednisolone and amoxicillin were among the APIs having the same identical names with several registration numbers. The inappropriate use of steroids (e.g., prednisolone), classified as “specially controlled drugs,” has been observed in the Thai community for an extended period [19]. Additionally, the misuse and overuse of antibiotics in Thailand, which causes antimicrobial resistance, is a major public health threat. Restricted measures, including registration system management, should be implemented to closely monitor and limit inappropriate use.

The second genre (615 brand names, 36.87% of total similarity results) was more complicated due to identical brand names derive from different manufacturers having the same APIs and possessed several registration numbers. As many as 438 pairs of registered brand names having same APIs, displayed two different registration numbers, and possessed by two different pharmaceutical companies or manufacturers. Generally, branding of individual pharmaceutical product bestows depends on a particular API of a pharmaceutical company or manufacturer to ensure authenticity [20]. For such identical brand names, having two or more registration numbers, and especially from numerous manufacturers, could cause confusion further leading to errors once prescribed or dispensed [21]. In Thailand, the pharmaceutical industrial landscape is somewhat perplexing. For example, a pharmaceutical company (distributor) might inherit a drug registration number for one formula and might have contracted the manufacturer to produce finished products for them. Pharmaceutical distributors and contract manufacturers establish their own traceability system [22]. However, the ownership of drug registration numbers ought to be determined whether pharmaceutical distributors or contract manufacturers should have exclusive possession. Once the possession of the drug registration number has been decided, the responsible authority should manage drug listings and registration directories systematically. Therefore, no other identical, repetitive, or similar brand names to ones that been registered previously, should not be allowed [22,23].

Paracetamol (500 mg tablet) was the generic name with the most registration number (96 registration numbers from 33 pharmaceutical companies). Surprisingly, all 96 registration numbers were registered as Paracetamol tablets^®^. Moreover, dexamethasone 0.5 mg and prednisolone 5 mg were registered by Dexamethasone tablets^®^ (57 registration numbers, 22 pharmaceutical companies) and Prednisolone tablet^®^ (48 registration numbers, 23 pharmaceutical companies), respectively. Nonproprietary names are suitable for use in pharmacopoeias, labeling, product information, advertisements, drug regulations, and scientific literatures [24]. Theoretically, brand names are distinctive, proprietarily protected, and exclusively employed by the owner [25]. Therefore, brand names for the pharmaceutical business should not be a repetition of, or similar to, the brand name used by another active licensee or a licensee whose license has been suspended or revoked for less than a full year [23]. Importantly, INNs should not be used to counterfeit brand names [26]. Since nonproprietary names are used as brand names in Thailand, though they have the same APIs, pharmaceutical excipients are certainly different. Different pharmaceutical excipients can cause allergic reactions. A similar situation occurred in India, where many pharmaceutical companies use brand names coined by INNs. The use of INNs to coin brand names can lead to confusion and may have important public health implications [26]. In the absence of comprehensive laws or policies regarding the use of INNs to imitate brand names and confusing brand names may still be part of Thailand’s medication complications.

Having multiple brand names derive from different manufacturers having the same APIs is a common scenario in many regions. However, in Thailand, it is complicated and very problematic since hospitals and private clinics normally dispense medication as pre-packs products with inadequate label e.g., no specific brand name, no lot numbers, no manufacturer name, no date of manufacture. Therefore, clearly specifying brand name is the only way to identify product efficiently. Then the findings of this study results are very impactful.

Although paracetamol has the highest number of registered brand names, it is the most commonly used over-the-counter drug, with a relatively broad therapeutic index. On the contrary, steroids (e.g., dexamethasone and prednisolone) are specially controlled drugs associated with increasing concerns of abuse and inappropriate use, and having many identical registered brand names might be a challenging circumstance to detect and specifically track the occurrence of medication complications. Routine checking or prevention of all such medication errors is improbable, especially if a thorough regulatory policy is never completed [27,28]

The last genre, single brand names from different manufacturers with diverse APIs, is limited and unthinkable. Four brand names (e.g., Myogesic^®^, Nizol^®^, Gemzil^®^, and Ferromin^®^) were identically used by different manufacturers for totally different APIs and had different registration numbers. The similarities between the brand names available in the market are unacceptable. If Thailand seeks to achieve a 2P safety goal, the awareness of LASA drug names should be intensively established from pre-marketing to post-marketing phase. Reducing problematic name pairs decreases the potential for confusion by healthcare professionals [4,5,28]. Collaboration and safety culture between the regulatory affair bodies and pharmaceutical manufacturers should be organized to build a common ground [12].

Drug safety should not be viewed only from the pre-market or post-market perspective, but rather as a continuum from the research and development, manufacturing, and registration processes until the medication use process [28,29]. Although the FDA established a strategy for systematic review and revision of drug registration in Thailand in 1997, no system or support mechanism has been clearly initiated [12,30,31]. Thailand’s drug registration system has been ineffective. For example, the periodic review every 5 years, instead of granting lifetime registration, was not as successful as expected. Additionally, the data management system for drug registration review was insufficient, as observed from the replication of identical brand names in this study.

Actually, the amount of registration number does not matter most as long as product can be identified correctly. But in Thailand as can clearly see in the results of this study that even the same brand name possesses more than 1 registration number and sometime having different APIs. Thus, the registration process should be designed to encourage both pharmaceutical manufacturers and regulatory authorities to determine whether the similarity in brand names are acceptable on the market. However, if pharmaceutical manufacturers decide to pursue more than one registration number for one formulation, brand name and physical character distinction of the product should be apparent. This may facilitate the Thai FDA’s anticipation of the actual number of registered drugs available on the market and their usage. Pharmaceutical distributors and contract manufacturers establish their own traceability system.

Possible recommendations of this study to the Thai FDA for improving the drug registration system to prevent brand name replication are as follows:

1. The Thai FDA should require that a product name and an assessment of the brand name be provided upon drug application submission. The assessment should determine whether the proposed brand name will be a LASA name with previously registered drug names. If so, it should be the responsibility of the manufacturer and registration regulators to resolve the problem prior to approval.
2. Periodic revision of drug registrations should be considered. A systematic review and revision should facilitate the cancellation of out-of-date or improper brand names and drive pharmaceutical manufacturers for continuous improvement.
3. Additionally, the exclusivity of drug registration possession should be settled between the manufacturer and distributor. Ideally, a brand name should be derived from a pharmaceutical company and have a single registration number.
4. The Thai FDA has the authority to grant or refuse a proposed brand name if it is misleading or has the potential for confusion with the name (brand or non-proprietary name) of other previously authorized products.
5. Registration database management systems should be updated accurately and regularly. An effective search function to compare similar or identical characteristics of drug products should be installed. The database should contain not only valid registration licenses but also those cancelled, withdrawn, or altered in any way. Moreover, systematic searches should address the consequences of medication errors due to LASA names. The registration database should be able to proactively identify any similarities in drug products and provide information for manufacturers to support decision making.

## Conclusions

The analogy results indicated that there were numerous tablets and capsules registered as “dangerous drug” and “specially controlled drug” in 2012 with identical brand names. These findings, revealing that identical brand names could be derived from the same manufacturers, APIs, dosage strengths, or otherwise, represent Thailand’s current state of drug registration difficulties. The results of this study offer recommendations to regulatory agencies to elevate registration procedures and mitigate the complications of LASA name errors and complications associated with the drug’s ongoing usage to ensure patient safety in the future.
